# Vapor phase polymerization of thieno[3,4-*b*]thiophene–tosylate and its application for dynamic structural coloration†

**DOI:** 10.1039/d4tc03789h

**Published:** 2025-03-12

**Authors:** Mohammad Shaad Ansari, Stefano Rossi, Giancarlo Cincotti, Renee Kroon, Magnus P. Jonsson

**Affiliations:** a Laboratory of Organic Electronics, Department of Science and Technology, Linköping University 601 74 Norrköping Sweden magnus.jonsson@liu.se; b Wallenberg Wood Science Center, Linköping University, Campus Norrköping, Norrköping Sweden

## Abstract

Conducting polymers are important for areas including energy storage, displays, sensors, nanooptics, and bioelectronics. Vapor phase polymerization (VPP) of conducting polymers can provide highly conductive homogenous thin films but was so far reported only for a limited number of materials. Here, we report VPP deposition of the low bandgap conducting polymer poly(thieno[3,4-*b*]thiophene):tosylate (pT34*b*T:Tos) and propose an application for dynamic structural coloration. Optimized films show high electrical conductivity of around 750 S cm^−1^, manifested optically as wide infrared absorption extending beyond 2000 nm. Electrochemical reduction reveals a neutral low bandgap peak around 1030 nm, making pT34*b*T comparably transparent also in its neutral state as opposed to other common conducting polymers. Moreover, the VPP process allows to spatially control the polymer properties and thickness *via* a UV exposure step before polymerization. We exploit this technique to create structurally colored images using the polymer as cavity spacer layer, locally varying its thickness and optical properties. We finally demonstrate dynamic tunability of structural colors based on the application of different potentials in an electrochemical cell.

## Abstract 2

We report vapor phase polymerization of the low bandgap conducting polymer poly(thieno[3,4-*b*]thiophene):tosylate (pT34*b*T:Tos) and explore its use for structural coloration, including UV patterned images and dynamic electrochemical tuning.

## Graphical abstract

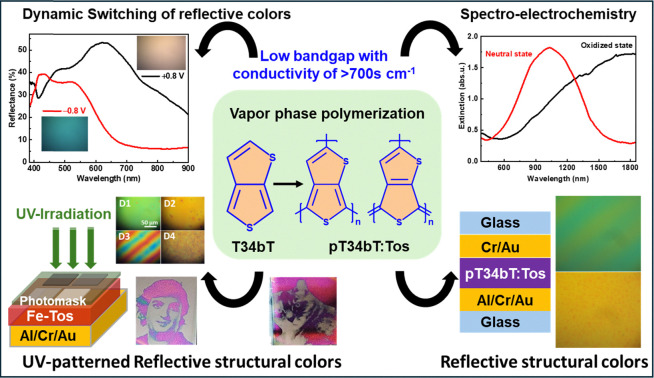

## Introduction

The electrical and optical properties of conducting polymers (CPs) can be tuned *via* their redox state, enabling concepts ranging from thin film transistors and organic capacitors to smart windows, dynamic optical metasurfaces, and reflective displays.^1–8^ The practicality of these conjugated materials relies on deposition of high-quality thin films on different substrates. In this respect, vapor phase polymerization (VPP) forms a versatile synthetic route to obtain particularly highly conductive films with controlled nanoscale thickness on any substrate.^6,9–14^ By contrast to electrochemical polymerization, VPP does not require a conductive substrate and it also enables convenient patterning at the micro- and macro-scales by UV exposure of the oxidant prior to polymerization.^15–18^ Examples of polymers shown to be compatible with VPP include polypyrrole, polyaniline, polythiophene and poly(3,4-ethylenedioxythiophene) (PEDOT).^19–24^ Those polymers were implemented for electrochromic displays as they exhibit a high optical contrast between different electrochemical states, due to the changes in optical absorption in the visible range.^18,25,26^ However, they are mostly suitable to reproduce two colors or for monochromatic displays. They are limited in terms of reproducing different colors also if combined with optical cavity effects^16^ due to the large optical absorption in the visible in their neutral state. A low-bandgap CP alternative is thienothiophene poly(thieno[3,4-*b*]thiophene) (pT34*b*T). It has previously been deposited by electrochemical or oxidative chemical polymerization,^27–30^ resulting in thin films with limited conductivity (typically below 5 S cm^−1^). The interest in pT34*b*T is related to its low bandgap, which stems from the energetically favored quinoid form^31,32^ and ability to form highly rigid polymer networks with extended π-conjugation. This marks a distinct difference for polymers using the fused ring of T34bT over polymers using standard thiophene.^33^ As a result, pT34*b*T films can be comparably transparent in both their doped and undoped states, because the neutral bandgap absorption is primarily in the near infrared (NIR) spectral range. This feature makes pT34*b*T distinct from other typical conducting polymers like PEDOT and polypyrrole, whose bandgap absorption peaks are in the visible region. We recently utilized the high visible transparency in both states to achieve redox-tunable structural colors with high brightness, based on electrodeposited pT34*b*T as a spacer layer in an optical cavity.^34^ The main working principle of those interferential cavities was a large thickness variation of pT34*b*T upon electrochemical actuation in an electrochemical cell. The concept provided full color tunability in the visible spectrum but also presented spatial inhomogeneities and limited lifetimes.

Here, we demonstrate VPP deposition of pT34*b*T and optimize the process by tuning polymerization conditions *via* temperature, time, and stoichiometry of the oxidant precursor. We focus on pT34*b*T deposited using Fe(iii) *p*-toluenesulfonate as dopant, which gives doped pT34*b*T films with tosylate as counterions (pT34*b*T:Tos). The optimized synthesis protocol provides thin films with record high room-temperature electrical conductivity of around 750 S cm^−1^. The redox state and doping level of the films could be reversibly controlled electrochemically, revealing a low bandgap in the neutral state around 1030 nm, hence enabling low absorption in the visible for all states. In accordance with VPP of other conducting polymers,^15,16^ we show that deposition can be altered by exposing the oxidant to UV light before polymerization. This affects both conductivity and thickness, which we use to produce structurally colored images by integrating the pT34*b*T film as spacer layer of an optical cavity. Finally, we demonstrate tunable structural colors by electrochemically varying the redox state of VPP-deposited pT34*b*T:Tos films on a metal mirror.

## Results and discussion

### Optimization of VPP conditions for deposition of pT34*b*T films

**Fig. 1:**
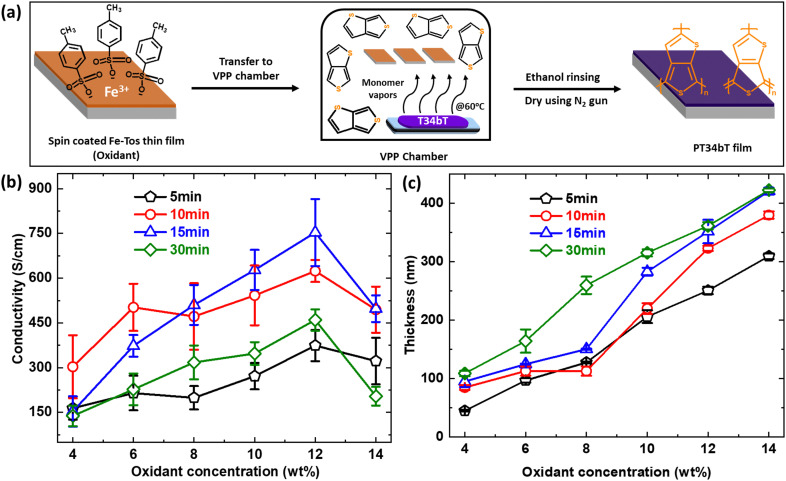
Oxidant concentration and vapor phase polymerization (VPP) time optimization. (a) Schematic representation of polymerization of T34bT using VPP synthetic route. (b) and (c) Variations of electrical conductivity and thickness of pT34*b*T:Tos with respect to different polymerization time and oxidant concentration. Error bars correspond to the standard errors of respective data set.

Fig. 1(a) depicts the process that we used for VPP deposition, which occurs at the liquid–vapor interface inside a heated vacuum chamber setup. As described in more detail in the experimental section, substrates were first coated with a Fe-Tos-based oxidant layer by spin coating. After drying, the substrates were exposed to monomer vapor inside a chamber under controlled reaction conditions, resulting in polymer thin film deposition. The procedure ends by a rinsing and drying step. To optimize the deposition process, we systematically varied oxidant concentration, reaction time and monomer vaporization temperature. Other variations would also be possible, including varying the type of oxidant. Here, we focus on Fe-Tos as oxidant since initial tests using Fe-Otf or FeCl_3_ showed clear deposition but mechanically unstable polymer films during the rinsing step. Fig. 1(b) and (c) shows the conductivity and thickness, respectively, of pT34*b*T:Tos films obtained for different oxidant concentrations (4–14 wt%) and polymerization times (5–30 min) while keeping the temperature of the monomer vaporization constant at 60 °C. We first note that we could successfully deposit high quality pT34*b*T films by VPP, with conductivity exceeding previous reports for pT34*b*T even for some of the less optimized process conditions.^30,35^ The electrical conductivity increased with increasing oxidant concentration up to 12 wt%, irrespective of polymerization time. The use of higher oxidant concentration (14 wt%) instead resulted in lower conductivity and higher surface roughness (Fig. S5, ESI†), with the same trend for all polymerization times. The film thickness increased monotonically with oxidant concentration for all polymerization times.

Comparing different polymerization times for the same oxidant concentration reveals a non-monotonic behaviour, with highest electrical conductivity at intermediate polymerization times of either 10 min or 15 min (depending on oxidant concentration). We also noticed that polymer films grown with short polymerization time exhibited poor substrate adhesion, sometimes resulting in patchy films. The longest polymerization time (30 min) also led to significantly lower conductivity (for 12% oxidant concentration the decrease was around 280 S cm^−1^ and 294 S cm^−1^ compared with 10 min and 15 min, respectively) compared with the intermediate times, and AFM analysis shows that longer polymerization time resulted in rougher polymer surfaces (Fig. S6, ESI†). We obtained the highest electrical conductivity of around 750 S cm^−1^ using an oxidant concentration of 12% and a polymerization time of 15 min. In contrast to the electrical conductivity, the film thickness increased monotonically with increasing oxidant concentration for all polymerization times (with exception for 8% oxidant concentration and 10 min polymerization time). For the higher oxidant concentrations, the thickness did not increase much further when increasing the polymerization time from 15 min to 30 min. Similar saturation effects have been observed for VPP of other conducting polymers.^36–38^

**Fig. 2:**
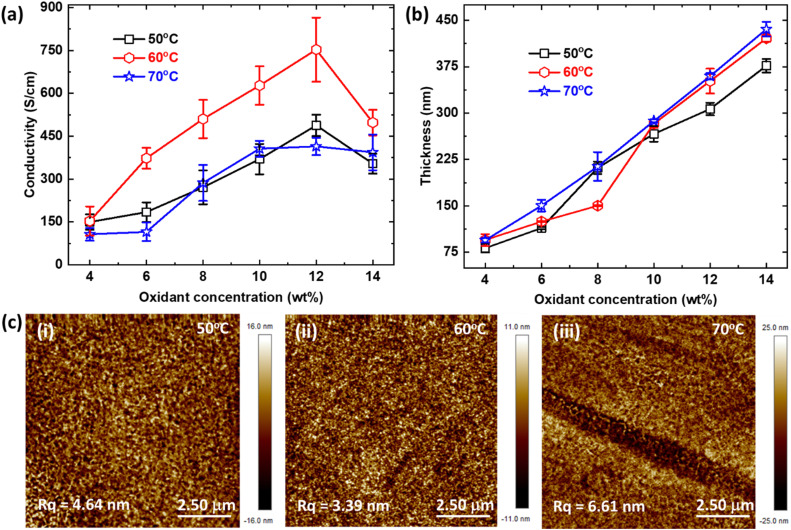
VPP temperature optimization. (a) Electrical conductivity of pT34*b*T films deposited at different oxidant concentrations and temperatures (polymerization time 15 min). (b) Measured thickness of the same films as in (a). The error bars in (a) and (b) correspond to the standard error of respective data set. (c) AFM images of T34bT:Tos thin films polymerized at temperatures of (i) 50 °C, (ii) 60 °C, and (iii) 70 °C (oxidant concentration of 12 wt%, 15 min VPP time). Calculated *R*_q_ values are indicated directly in the AFM images.

Next, we study the role of the temperature used to vaporize the monomer on the formation and electrical properties of pT34*b*T films. Fig. 2(a) and (b) show the change in pT34*b*T electrical conductivity and thickness, respectively, as functions of the vaporization temperature (for a fixed polymerization time of 15 min). For all oxidant concentrations except the lowest, the conductivity was higher for polymerization using 60 °C compared with both the lower and higher vaporization temperatures. At the optimum oxidant concentration of 12%, the conductivity dropped from around 750 S cm^−1^ using 60 °C to values around 400–450 S cm^−1^ when increasing or decreasing the temperature by 10 °C. This significant variation (around 300 S cm^−1^) highlights the important role of the vaporization temperature to obtain optimized polymerization kinetics in terms of charge transport properties. Interestingly, we found smaller variations in polymer film thickness upon varying the temperature. At 12% oxidant concentration, the thickness was almost the same for 60 °C and 70 °C while it decreased by around 50 nm for 50 °C (around 15% decrease). In general, polymerization at 70 °C led to thickest films for all oxidant concentrations, which agrees with more rapid supply of monomer to the oxidant film. At the optimum 12% oxidant concentration, the thickness for 50 °C reaction temperature was about 25% lower than at 70 °C, while deposition at 60 °C led to almost as thick samples as for 70 °C. At first sight, the latter may indicate that the deposition rates for 60 °C and 70 °C were similar. However, this may be an artifact from the deposition reaching saturation, similar to the fact that we did not obtain larger thicknesses when increasing the polymerization time from 15 min to 30 min for the highest oxidant concentrations (Fig. 1(c)). Hence, the polymerization kinetics at 60 °C and 70 °C may still be different and responsible for the strong variation in conductivity. Focusing on the optimum oxidant concentration of 12%, we also note that polymerization at 60 °C resulted in more smooth films. Atomic force microscope (AFM) images reveal root mean square roughness (*R*_q_) of around 3.4 nm using 60 °C, while the values were 4.6 nm and 6.6 nm using 50 °C and 70 °C, respectively (Fig. 2(c)). To summarize, the optimized parameters for highest conductivity are: oxidant concentration of 12 wt%, 15 min VPP time and vaporizing temperature of 60 °C. These conditions reproducibly resulted in pT34*b*T films with conductivity exceeding 700 S cm^−1^. The combined results regarding thickness and conductivity upon varying oxidant concentration, VPP time and VPP temperature are tabulated in Table 1.

**Table 1:** Optimization table for vapor phase polymerization process of pT34*b*T:Tos

S. no.	Fe-Tos wt%	Temperature (°C)	Time (min)	Thickness (nm)	Conductivity (S cm^−1^)
1	4	60	5	45	164
2	6	60	5	97	214
3	8	60	5	128	199
4	10	60	5	206	271
5	12	60	5	250	374
6	14	60	5	309	322
7	4	60	10	85	303
8	6	60	10	113	502
9	8	60	10	112	471
10	10	60	10	219	542
11	12	60	10	323	624
12	14	60	10	379	494
13	4	60	15	94	153
14	6	60	15	124	371
15	8	60	15	150	510
16	10	60	15	282	627
**17**	**12**	**60**	**15**	**351**	**753**
18	14	60	15	420	497
19	4	60	30	109	138
20	6	60	30	164	227
21	8	60	30	260	317
22	10	60	30	315	347
23	12	60	30	362	459
24	14	60	30	422	204
25	4	50	15	81	149
26	6	50	15	114	184
27	8	50	15	211	271
28	10	50	15	266	370
29	12	50	15	306	487
30	14	50	15	376	354
31	4	70	15	95	106
32	6	70	15	150	115
33	8	70	15	214	286
34	10	70	15	287	406
35	12	70	15	360	414
36	14	70	15	436	393

### *In situ* spectro-electrochemical measurements for VPP pT34*b*T:Tos films

**Fig. 3:**
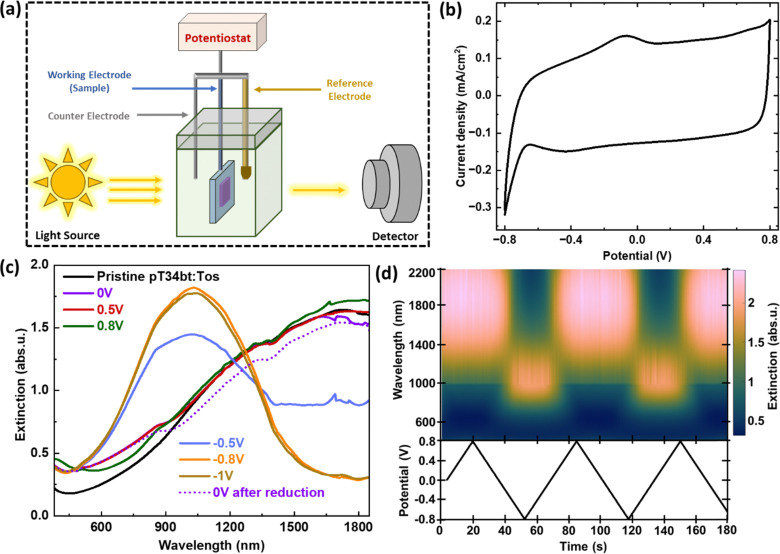
Spectro-electrochemical switching of VPP pT34*b*T:Tos. (a) Schematic representation of the experimental setup. (b) Stabilized CV curve of a VPP pT34*b*T:Tos film on ITO-coated glass, using acetonitrile based LiClO_4_ electrolyte and Ag/Ag^+^ reference electrode at a scan rate of 50 mV s^−1^. (c) Extinction spectra at different electrochemical potentials for the same experiment as in (b). (d) Extinction color map related to electrochemical state variation of the polymer during dynamic CV analysis.

After demonstrating successful VPP of highly conducting pT34*b*T:Tos films, we performed *in situ* spectro-electrochemical measurements to determine effects of varying their redox state, (Fig. 3(a)). Electrochemical tuning was performed for pT34*b*T:Tos films deposited on ITO-coated glass, using 0.1 M LiClO_4_/acetonitrile electrolyte and a 3-electrode setup with a Ag/Ag^+^ reference electrode. We choose this electrolyte as it provides low absorption both in the visible and the NIR regions. Fig. 3(b) presents a cyclic voltammogram (CV) of a VPP pT34*b*T thin film (using the optimized conditions from above), recorded in the potential window of −0.8 to 0.8 V with 50 mV s^−1^ scan rate. Besides a noteworthy rectangular-shaped capacitive contribution, the CV results revealed an oxidation peak at around −0.07 V and a reduction dip at around −0.43 V. Fig. 3(c) presents corresponding extinction spectra at different voltages. We first note that the pristine material (before applying any potential) shows low absorbance in the visible region and gradually increased absorption in the NIR region. This agrees with the high conductivity and corresponding broad infrared absorption of mobile polaronic carriers in the deposited polymer film.^39–41^ The extinction spectrum at zero voltage was similar to the pristine film but with slightly lower NIR absorbance (decrease of ∼0.10 abs. u.). Increasing the oxidation potential to 0.5 V and further to 0.8 V slightly increased (increase of ∼0.08 abs. u.) the polaronic NIR absorbance, implying further increased doping level. Applying a reduction potential (−0.5 V) weakened the NIR polaronic absorption and induced a new peak centered at a wavelength around 1030 nm. These effects originate from lowering of the charge carrier density by de-doping and transition of the polymer to its neutral state. The neutral peak at 1030 nm (1.20 eV) confirms the low bandgap of the material. Further incrementing the reduction potential (−0.8 V) completely depleted the polaronic feature and strengthened the neutral bandgap peak. The response then saturated and only showed minor difference if increasing the reduction potential from −0.8 V to −1 V. Subsequently applying 0 V after the reduction process returned the optical response to the original response at 0 V, with attenuation of the neutral peak and reappearance of the NIR polaron band. This demonstrates reversible switching, which is corroborated from the temporal variation of the extinction over several cycles (Fig. 3(d)). The results thereby demonstrate that VPP pT34*b*T:Tos film can be reversibly tuned between its oxidized and reduced forms by the application of moderate bias potentials, with absorption centered in the NIR for all electrochemical states.

### Reflective structural colors using pT34*b*T:Tos optical cavities

**Fig. 4:**
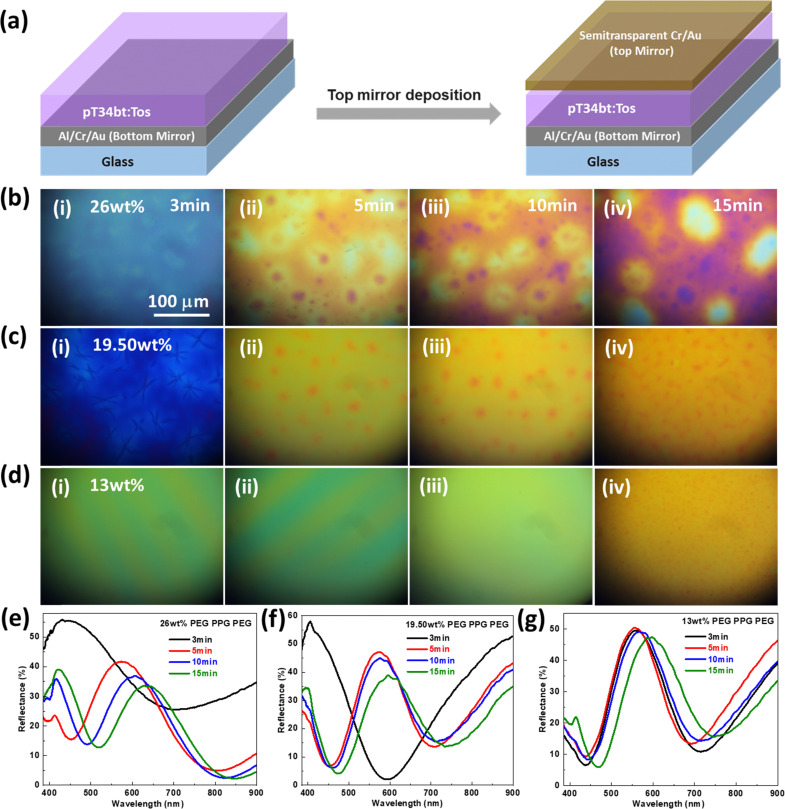
Reflective structural colors based on pT34*b*T:Tos optical cavities. (a) Schematic illustration of fabrication route for an optical cavity. (b)–(d) (i)–(iv) Microscopic images and (e)–(g) represent the reflectance spectra of VPP pT34*b*T:Tos based optical cavities with the different concentration of copolymer (PEG–PPG–PEG) 26 wt%, 19.50 wt% and 13 wt% and polymerization reaction time (3 min, 5 min, 10 min and 15 min), which leads to thickness change of pT34*b*T:Tos polymer films and able to generate the different reflective structural colors.

The low visible absorption of the polymer is promising for use as spacer layer in optical cavities that produce structural colors.^34^ We here focus on a cavity design where the pT34*b*T film is sandwiched between a bottom mirror (70 nm Al/3 nm Cr/7 nm Au) and a semitransparent top layer (3 nm Cr/7 nm Au, Fig. 4(a)). The inclusion of Cr in the top layer changes the response from that of a traditional Fabry–Pérot cavity by adding broadband absorber cavity effects. We previously showed that this can lead to high-chromaticity reflective colors *via* the formation of distinct reflection peaks instead of reflection dips.^42^ Finite element simulations confirm this behavior for cavities with pT34*b*T:Tos as spacer layer and illustrate the expected dependence on the polymer thickness (Fig. S9, ESI†). Experimentally, we varied the pT34*b*T spacer thickness *via* the VPP time and obtained clear reflective colors (Fig. 4(b)). However, we also noticed non-homogeneous effects across the surfaces. This behavior may be related to the concentration of water in the oxidant layer which is related to the hygroscopic copolymer (PEG–PPG–PEG). While a certain amount of water is beneficial for the polymerization process, too high concentration can lead to the formation of crystallites and corresponding non-homogeneity of the final material.^43–45^ We therefore repeated the procedure using lower concentration of copolymer (26 wt% [original], 19.50 wt% and 13 wt%) as well as for different VPP times (3, 5, 10 and 15 min).

From microscopy images (Fig. 4(c) and (d)), we observe that the structural defects decreased notably with the decrease in copolymer concentration, with the lowest concentration resulting in most homogeneous colors. As expected, the reflective colors varied with polymerization time due to variations in polymer thickness. The color control appears as gradual red-shift of the reflection peak position for longer polymerization times (Fig. 4(e)–(g)). The peak shift was largest for the highest copolymer concentration, revealing a shift in reflectance peak position from 450 to 630 nm. The color control was less strong for lower concentrations, implying the need for a compromise between homogeneity and color control. Fig. 4(f) and (g) show that cavities with shortest polymerization times possessed highest peak reflectance (around 50–60% depending on copolymer concentration). The peak reflectance then decreased with increasing polymerization time, to about 30% if using the highest copolymer concentration while it remained close to 50% for the lowest copolymer concentration. Simulation results validate the trend of decreasing peak reflectance for cavities with increasing thickness (Fig. S9, ESI†).

### Reflective structural colors utilizing UV-irradiated pT34*b*T:Tos optical cavities

**Fig. 5:**
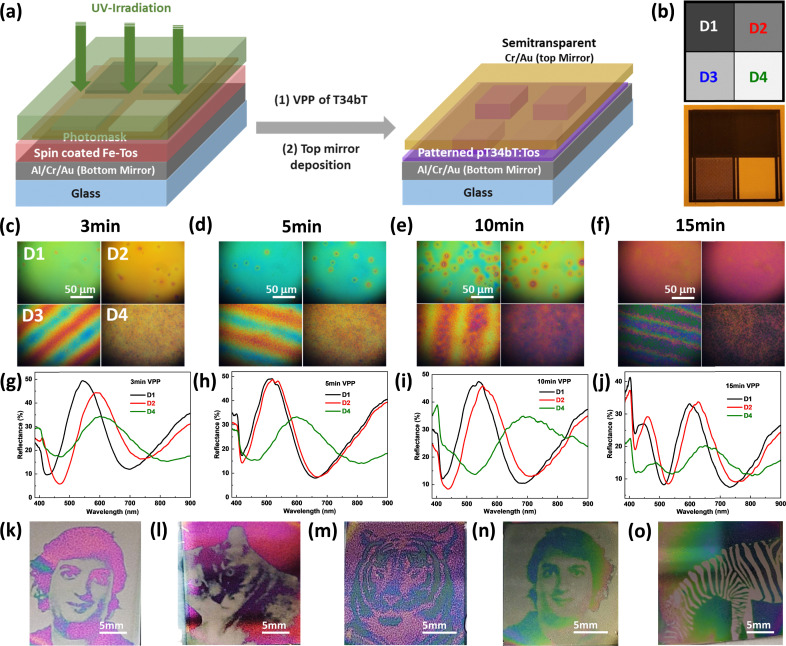
Reflective structural colors based on UV-irradiated pT34*b*T:Tos optical cavities. (a) Graphical illustration of fabrication of UV-patterned optical cavity-based device. (b) Fabricated grayscale patterns having different pixels (D1–D4) and digital photograph of printed grayscale mask. (c)–(f) Microscopic images of UV-irradiated (20 min) pT34*b*T:Tos based optical cavities using the printed grayscale mask with different VPP times (3 min, 5 min, 10 min and 15 min). (g)–(j) Reflectance spectra of same optical cavities with different VPP times. (k)–(o) UV-patterned pT34*b*T based images with 20 min UV exposure and different concentration of PEG–PPG–PEG 26 wt% for panel (k)–(m) while 19.50 wt% for panel (n) and (o) using different grayscale photomasks, revealing the possibility to generate the UV controlled structurally colored images. Scale bars are included in the respective images.

A different way of controlling thickness of VPP-deposited polymer films is to expose the oxidant layer to UV light before polymerization. Previous works on PEDOT showed that UV exposure modulates the polymerization kinetics, resulting in polymer films that increases gradually in thickness with increasing UV dose.^15^ Exposure through a mask to locally control the UV dose then enables high-resolution patterns and optical gradients to be produced in a convenient manner.^16,17^ Edberg *et al.* reported that exposure of UV light changes the Fe(iii) coordination shell in the oxidant solution, leading to faster polymerization kinetics which results in the synthesis of shorter polymer chains with different electronic structure and morphology compared to films without the UV light exposure step.^15^ Moreover, their FTIR spectra for UV-irradiated and non-irradiated FeTos oxidant films showed a significant change in the peak appearing at about 3400 cm^−1^, which corresponds to free OH groups. Due to presence of bonded water molecules in the oxidant layer, the ionic mobility of the oxidant may get influenced by the change in the coordination of water. Changes in the coordination between iron, tosylate, and water have been shown to have large effects on polymerization kinetics.^46^ Our tests using a photomask with areas of different UV transmittance (D1–D4, see Fig. 5(b), with digital picture of same mask) confirms that the concept of UV patterning works for VPP pT34*b*T:Tos, manifested as significant increase in film thickness (roughly 3 times for D4) and decrease in electrical conductivity with increasing UV exposure dose (Fig. S10, ESI†). The UV transmittance at 365 nm for D1, D2, D3, and D4 were 0.25%, 1.20%, 47.38%, and 65.81%, respectively.

Fig. 5(a) illustrates how we explored the concept to create UV-patterned nano-optical cavities (using the same cavity configuration as above). In brief, the oxidant (12 wt% oxidant, 13 wt% copolymer) on the bottom mirror was exposed to UV light for 20 min using the four-area photomask. This was followed by VPP deposition and thermal evaporation of the top metal layer to complete the cavities (see Methods for details). Fig. 5(c)–(f) display reflective microscopy images of final samples for different VPP times. For all tested VPP times, the different areas (different UV exposure doses) on the same sample led to different colors. This is primarily attributed to the modulation of the polymer thickness but variations in optical properties may also contribute.^16^Fig. 5(g)–(j) present corresponding reflectance spectra from pixels D1, D2 and D4 for different VPP times. We observed clear peak redshift of the reflection peak with UV exposure dose, corroborating the increase in film thickness. Pixel D3 was not included in Fig. 5(g)–(j) because results for D3 presented gradually varying colors across the surfaces in a line shape. This effect is attributed to the photomask not being homogeneous at the microscale but containing lines that will spatially modulate the UV transmittance (Fig. S11a, ESI†). As confirmed by profilometry (Fig. S11b–e, ESI†), this creates a spatial thickness modulation that explains the observed color gradients. This explanation also agrees with the periodicity of the lines being the same regardless of VPP time. Unintentionally, the UV patterning results for pixel D3 thereby demonstrate the possibility to create structurally colored gradients by locally controlling the UV transmittance of the photomask. Fig. 5(k)–(o) exemplify this concept for more elaborate patterns and images (digital images captured using Samsung S20 mobile phone). We generated these colorful images using greyscale shadow masks, on 2.5 × 2.5 cm^2^ metal-deposited glass slides. We used 26 wt% (panels (k)–(m)) and 19.50 wt% (panels (n) and (o)) of copolymer in the oxidant precursor since results for 13 wt% of copolymer were less mechanically stable and did not lead to well resolved images.

### Dynamically electrochemical actuation of reflective structural colors based on pT34*b*T:Tos deposited on a metal film

**Fig. 6:**
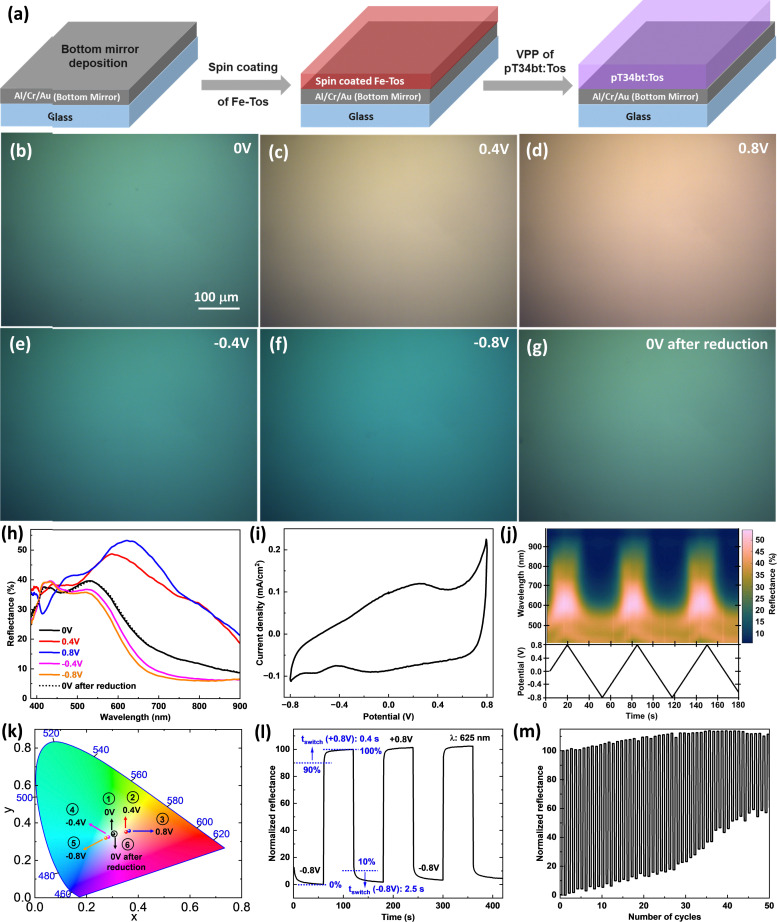
Electrochemical actuation of reflective structural colors based on pT34*b*T:Tos deposited over metal surface. (a) Schematic representation of device used for electrochemical actuation. (b)–(g) Microscopic images of tuned structural color based on pT34*b*T:Tos over bottom mirror (70 nm Al/3 nm Cr/7 nm Au) with the application of different electrochemical potentials. (h) Reflectance spectra, showing the tuning of the reflectance peak with different positive and negative electrochemical potential. (i) Cyclic voltammogram (CV) curve of VPP pT34*b*T:Tos on metallic mirror using aqueous NaDBS electrolyte medium, Ag/AgCl reference electrode at the scan rate of 50 mV s^−1^. (j) Reflectance color map (top) during dynamic variation of the applied potential (bottom). (k) CIE 1931 diagram obtained *via* conversion of reflectance data (from panel (h)) to CIE coordinates. (l) Normalized reflectance (at a wavelength of 625 nm) for same device upon periodic changes of an applied potential (±0.8 V) for a few cycles (60 s duration for each step) with the extracted switching times for oxidation (+0.8 V, 0% to 90% change) and reduction (−0.8 V, 100% to 10% change) potentials. (m) Long-term stability test of the same device as in panel (b) upon prolonged measurements of periodically changing the applied potential (±0.8 V).

Many applications of reflective colors would benefit from dynamic tunability, for example aiming at electronic readers in color with high brightness. Here, we adopted a different structure without top metal layers to ensure good contact between the polymer and an electrolyte used for redox-switching (Fig. 6(a)). Chen *et al.* employed the same device structure based on PEDOT:Tos for *in situ* electrochemical structural color tuning, but observed limited color variation and limited reflectance across all electrochemical states.^16^ We further used 12 wt% oxidant, 13 wt% of copolymer and 3 min VPP time to deposit the pT34*b*T:Tos films on metallic substrates. Fig. 6(b)–(g) present microscope reflection images at different applied potentials (−0.8 V to +0.8 V using aqueous NaDBS electrolyte and Ag/AgCl reference electrode). Fig. 6(h) presents the corresponding redox-tunable reflection spectra, revealing significant variations in reflectance with changes in potential. In particular, the application of an oxidation potential (positive potential) led to the emergence of a reflection peak that red-shifted with increasing potential. The total shift was close to 200 nm and connected to color changes, from turquoise green at −0.8 V, to yellowish-green at 0 V and further to pale orange for +0.8 V. The magnitude of the reflectance at the peak also increased during the red-shift, to above 50% at +0.8 V. Monitoring the reflectance spectrum during dynamic electrochemical state variation reveals reversible switching over several cycles (Fig. 6(j)). The results are consistent with reversible swelling during redox tuning, which was also observed for electropolymerized pT34*b*T:DBS using the same electrolyte although with opposite behaviour (red-shifting with positive potentials instead of negative).^34^ We converted the dynamic switching reflectance spectra (Fig. 6(h)) at different potentials into CIE coordinates and plotted them on the CIE 1931 diagram to illustrate the dynamic structural color changes within the visible spectrum (Fig. 6(k)). In the CIE diagram, we observed a color transition during the application of positive potentials: from yellowish-green (0 V, CIE coordinates: 0.3047, 0.3402) to pale yellow (0.4 V, CIE coordinates: 0.3534, 0.3511) and eventually to orange (0.8 V, CIE coordinates: 0.3653, 0.357). During the application of reduction potentials, the colors shifted to pale yellowish-green (−0.4 V, CIE coordinates: 0.2851, 0.3239) and turquoise green (−0.8 V, CIE coordinates: 0.2746, 0.3186). Notably, after applying a reduction potential, the electrochromic device returned to its initial yellowish-green state at 0 V (CIE coordinates: 0.3073, 0.341). These results demonstrate that the electrochromic device undergoes a reversible color change, shifting from yellowish-green to orange during oxidation and transitioning to turquoise green during reduction. Fig. 6(l) shows the variation in normalized reflectance at a wavelength of 625 nm upon the application of oxidation (+0.8 V) and reduction (−0.8 V) potentials for multiple cycles. The plot demonstrates reversible changes in the reflectance for each change in the applied bias potential. We have extracted the switching times considering 0% to 90% of the initial and final reflectance values for oxidation (+0.8 V) and 100% to 10% for reduction (−0.8 V) potentials. The switching time for reflectance changes during oxidation was about 0.4 s, suggesting quite fast switching while the switching time upon reduction was around 2.5 s. Fig. 6(m) represents the long-term stability of the same electrochromic device upon prolonged cycling. We observed that the device exhibited no significant change in the reflectance modulation during the initial cycles. The device showed less than a 10% decrease in the modulation of the reflectance during the first 30 cycles of varying the potential between −0.8 V and +0.8 V, while the change in the reflectance decreased by approximately 40% after around 50 cycles.

## Conclusions

In summary, we have demonstrated VPP deposition of pT34*b*T:Tos and provided a systematic optimization of polymerization conditions based on polymerization time, temperature and oxidant concentration. The most conducting pT34*b*T:Tos films show values around 700 S cm^−1^ at room temperature, which to our knowledge is considerably higher than previous reports on pT34*b*T. Electrochemical reduction to the polymer's neutral state reveals a low bandgap around 1030 nm, indicating low visible absorption in both electrochemical states. The electrochemical switching was reversible, as explored for dynamically tunable structural colors. The copolymer content in the oxidant was modified to reduce structural defects and to obtain more homogeneously colored films. We further showed that the VPP process is compatible with the concept of UV patterning to spatially modulate the thickness along the surface, here explored for structurally colored cavities using the polymer as spacer layer. Vivid and highly resolved color images could be generated using UV exposure through grayscale photomasks. In turn, structurally colored images and tunable nanooptical cavities may aid the development of several important application, including backlight-free energy-efficient reflective displays in color.

## Experimental section/methods

### Materials

The chemicals involved in the polymerization procedure and device fabrication were of analytical grade and used without any purification. Clevios CB 54 V3 (54% wt/wt Fe(iii) *p*-toluenesulfonate (Fe-Tos) in butanol) was procured from Heraeus (Germany). Triblock copolymer; poly(ethylene glycol)-*block*-poly(propylene glycol)-*block*-poly(ethylene glycol) PEG–PPG–PEG (average *M*_n_ ≈ 5800) and T34bT monomer (140.23 g mol^−1^) were purchased from Sigma-Aldrich and Accela respectively.

### Vapor phase polymerization (VPP) and device fabrication for electrochemical switching

First, microscope glass slides or ITO-coated glass substrates (2.5 × 2.5 cm^2^) were cleaned *via* subsequent steps of ultrasonication in a 2% soap (Hellmanex) solution in deionized (DI) water, DI water, acetone, DI water and isopropanol for 10 min, for individual cleaning step. All substrates were then dried using nitrogen gun, followed by oxygen plasma treatment for 5 min at 100 W, before starting the polymerization procedure. pT34*b*T:Tos thin films were deposited on cleaned substrates using VPP according to the following procedure. Oxidant solution for T34bT VPP was prepared by dissolving different weight % (wt%) of Fe(iii) *p*-toluenesulfonate (Fe-Tos; 4 wt%, 6 wt%, 8 wt%, 10 wt%, 12 wt% and 14 wt%) and triblock copolymer PEG–PPG–PEG (26 wt%; same amount for all oxidant solutions) in ethanol (99.7%, Solveco). The oxidant was spin-coated onto the pre-cleaned substrates at 1500 rpm spinning speed for 30 s, followed by thermal annealing at 70 °C for 1 min. The substrates were then transferred to a heated vacuum chamber (Vacuo-temp (SELECTA)) to proceed with VPP of T34bT. 10 μL of T34bT was drop-casted onto a separate glass substrate and placed inside the polymerization chamber. We varied the temperature used to vaporize the monomer (50 °C, 60 °C and 70 °C) and time (from 3 to 30 min) and used a pressure of 70 mbar. The samples were kept on glass slides on a plastic stand inside the chamber, as shown in Fig. S1 (ESI†). Washing was done after polymerization to remove unreacted Fe(ii) tosylate residues, loosely adsorbed monomer and byproducts, by submerging the pT34*b*T samples in ethanol in a Petri-dish for around two hours, followed by gentle drying using a nitrogen gun.

### Fabrication of bare and UV-patterned optical cavities for reflective structural colors

To grow pT34*b*T films on mirrors, we deposited 70 nm Al on precleaned glass substrates (10–15 Å s^−1^), followed by 3 nm Cr (0.5 Å s^−1^) adhesion layer and 7 nm Au (2–5 Å s^−1^), using thermal evaporation (PVD handy 2T, Vaksis). The substrates with metal films were then treated with UV-ozone for 15 min before polymerization. pT34*b*T:Tos films were deposited on metallic films using VPP method as described above, with thickness varied by the polymerization time. To make complete optical cavities, we deposited a semitransparent mirror (3 nm Cr/7 nm Au) on top of the pT34*b*T:Tos film. For UV patterning experiments, the oxidant films on metallic mirrors were irradiated by a UV lamp through photomasks using a mask aligner, at wavelength of 365 nm and power of 10 mW cm^−2^ for 20 min. Grayscale photomasks (took prior permission from one of co-author) were printed using an office inkjet printer (RICOH, Japan) on transparent plastic films. After UV exposure, substrates were transferred to the vacuum chamber for deposition of pT34*b*T:Tos by VPP.

### Electrical, topographical, optical and spectro-electrochemical characterization

The conductivity of the VPP pT34*b*T:Tos thin films (2.5 × 2.5 cm^2^) was estimated *via* a four-point probe set-up using a Keithley 2400 and a Signatone Pro4 S-302 resistivity stand and calculated *via σ* = 1/(*R*_s_ × *t*), where *σ* is electrical conductivity, *R*_s_ is sheet resistivity and *t* is the thickness of VPP film. We measured the resistance at several places of film to estimate the statistical errors. The geometrical correction factor was calculated *via* considering the substrate dimensions, point spacing and placement of four-point probe on the film, which was required to estimate the *R*_s_ from the measured resistance values. Dektak3ST Veeco profilometer was used to determine the ‘*t*’. The VPP thin films were scratched using a sharp knife to measure the film thickness at the exposed film edge. The thickness and electrical conductivity were determined as the average between measurements at several positions for each film. Surface topography of the VPP thin films was analyzed using Veeco Dimension 3100 AFM and Nanoscope Analysis 1.8 software. UV-vis-NIR extinction spectra were measured using Lambda 900, PerkinElmer Instruments in the wavelength range of 300–3300 nm with a scan rate of 5 nm s^−1^.

Reflectance spectra were obtained using a customized setup based on a microscope equipped with a spectrophotometer. In brief, the devices were irradiated using a 50 W halogen lamp of an optical microscope (Nikon Eclipse L200 N, Japan). At the camera port, reflected light was split into two portions using a beam splitter (SM1CP2, Thorlabs), with one portion used for image generation and the other for recording spectra through an optical fiber (50 μm diameter, Thorlabs M14L05) and a spectrometer (QePRO, Ocean Optics, USA). Some digital images were captured using a Samsung S20 cellphone.

Spectro-electrochemical characterization was performed using a customized electrochemical setup (Back microscopy EFC, Redox.me) in a three-electrode system. Platinum coil (0.6 mm diameter and 250 mm long wire) was used as the counter electrode. All electrochemical experiments were conducted using SP-200, Biologic and Autolab PGSTAT204, Metrohm potentiostat. 0.1 M LiClO_4_ in acetonitrile or 0.1 M aqueous NaDBS were used as electrolytes. The area of the sample exposed to the electrolyte was around 1.33 cm^2^. Cyclic voltammetry and chronoamperometry were performed for electrochemical switching as well as for recording the optical response. Spectro-electrochemical experiments were carried out using the same setup which was used to measure reflectance spectra. We used a 50 μm diameter optical fiber with 100 ms integration time to connect a reflectance microscopy (50× objective) to spectrophotometer. Moreover, the motivation behind choosing this small diameter optical fiber was to get assured to record the maximum possible signals from the small active area. Microscopic image and optical response were captured simultaneously with the same focal plane of the output fiber order. We normalized all reflectance spectra with respect to Ag Mirror (Thorlabs).

## Author contributions

Mohammad Shaad Ansari: conceptualization, data curation, formal analysis, investigation, methodology, visualization, writing – original draft. Stefano Rossi: methodology, data curation, formal analysis, writing – review & editing. Giancarlo Cincotti: methodology, writing – review & editing. Renee Kroon: writing – review & editing. Magnus P. Jonsson: supervision, formal analysis, funding, writing – review & editing.

## Data availability

The data are available *via* Zenodo a https://doi.org/10.5281/zenodo.14918529.

## Conflicts of interest

All authors declare that they have no conflicts of interest.

## Supplementary Material

TC-013-D4TC03789H-s001
