# Supplementary material for: Vapor phase polymerization of thieno[3,4-b]thiophene–tosylate and its application for dynamic structural coloration
Source: J Mater Chem C Mater. 2025 Mar 12;13(15):7643–53. doi: 10.1039/d4tc03789h (PMC11898598; doi:10.1039/d4tc03789h)
Supplement: TC-013-D4TC03789H-s001 [file TC-013-D4TC03789H-s001.pdf]

Supplementary Information (SI) for Journal of Materials Chemistry C.
This journal is © The Royal Society of Chemistry 2025

Electronic supplementary information

Vapor Phase Polymerization of Thieno[3,4-b] thiophene-Tosylate and its Application for Dynamic Structural Coloration

Mohammad Shaad Ansari[1], *Stefano Rossi*[1], *Giancarlo Cincotti*[1], *Renee Kroon*[1] *and Magnus P. Jonsson*[1]*

[1]Laboratory of Organic Electronics, Department of Science and Technology, Linköping University, 601 74 Norrköping, Sweden

E-mail: magnus.jonsson@liu.se

Figure S1: Digital image of VPP set-up

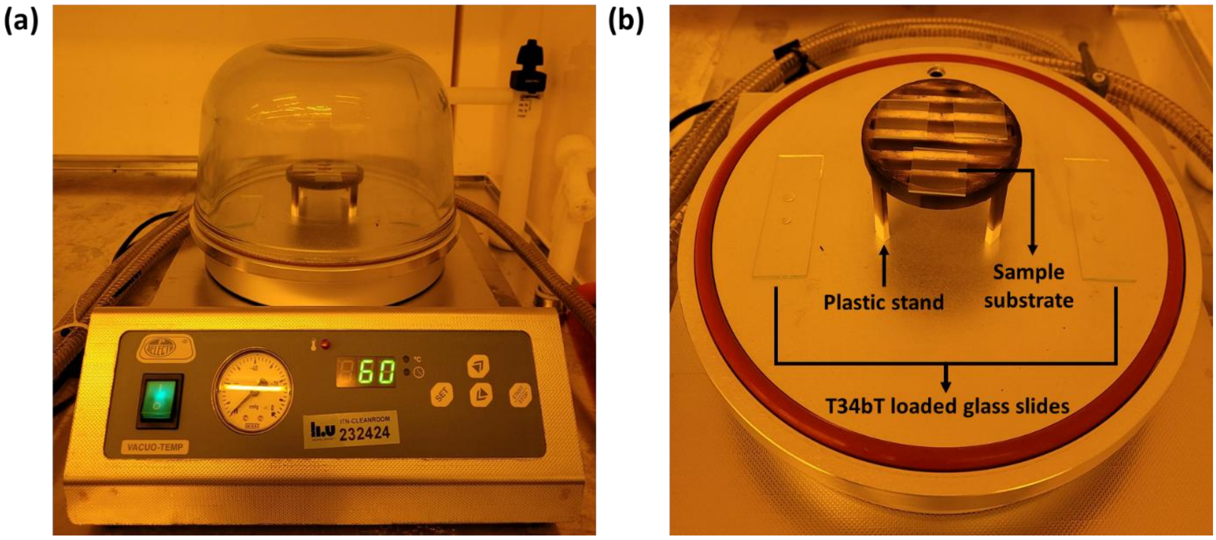

Figure S1: (a) Digital image of the setup used for the VPP process. (b) Arrangement of sample substrates on a plastic stand (avoiding direct contact with the hot plate) while glass slides with T34bT monomer are in direct contact with the hot plate.

Figure S2: AFM analysis of 12 wt% Fe-Tos based pT34bT:Tos film

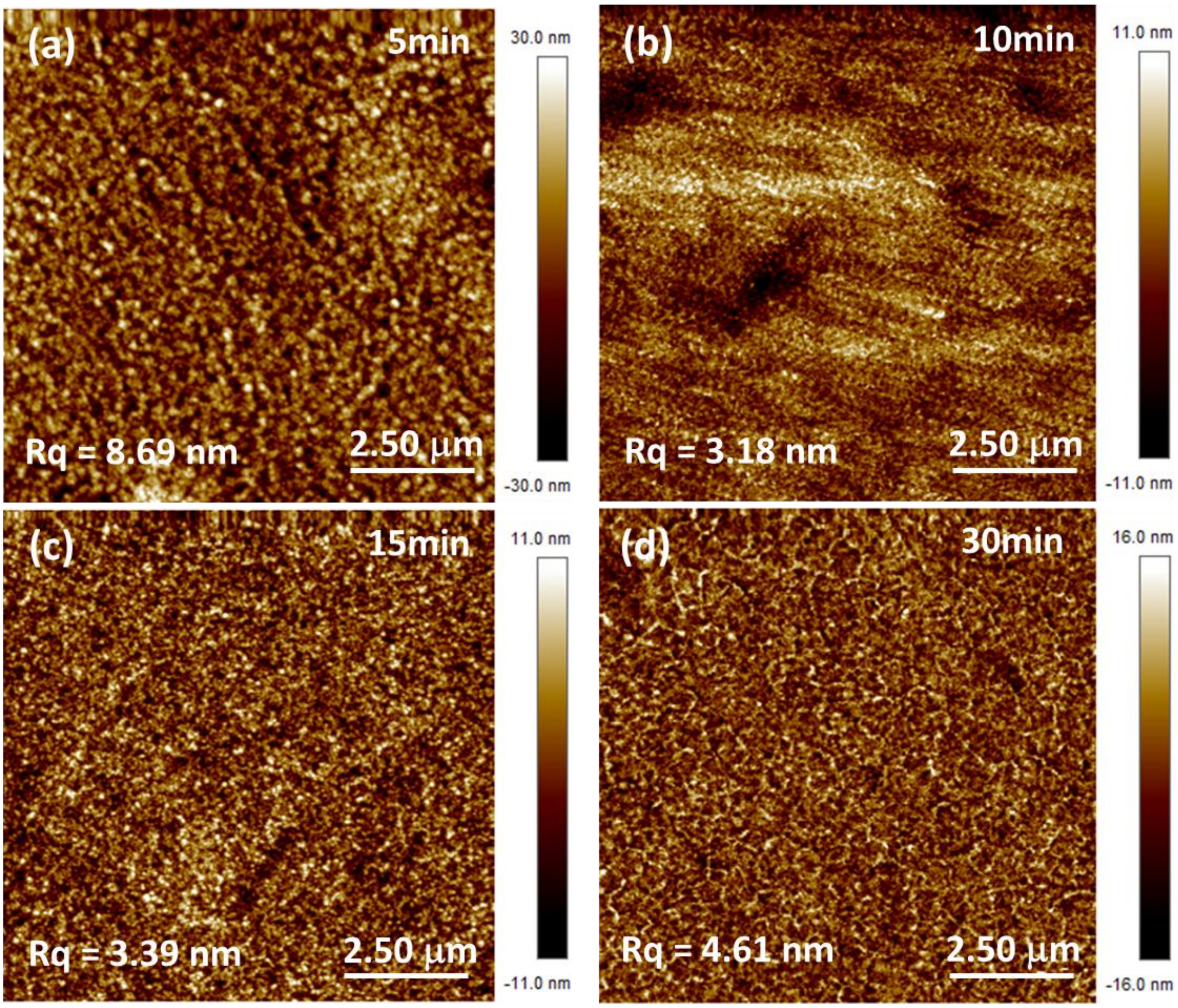

Figure S2: (a) AFM image of VPP pT34bt:Tos thin film with 12 wt% Fe-Tos and 5 min VPP time. (b) AFM image of same VPP pT34bt:Tos thin film with 10 min VPP time. (c) AFM image of same VPP pT34bt:Tos thin film with 15 min VPP time. (d) AFM image of same VPP pT34bt:Tos thin film with 30 min VPP time. AFM images show the topological features and roughness values (Rq) are indicated in respective AFM image.

Figure S3: Vapor phase polymerization, absorption profile and topological features of VPP time '5 min' based pT34bT:Tos film with various oxidant concentration and VPP temperature of 60 °C

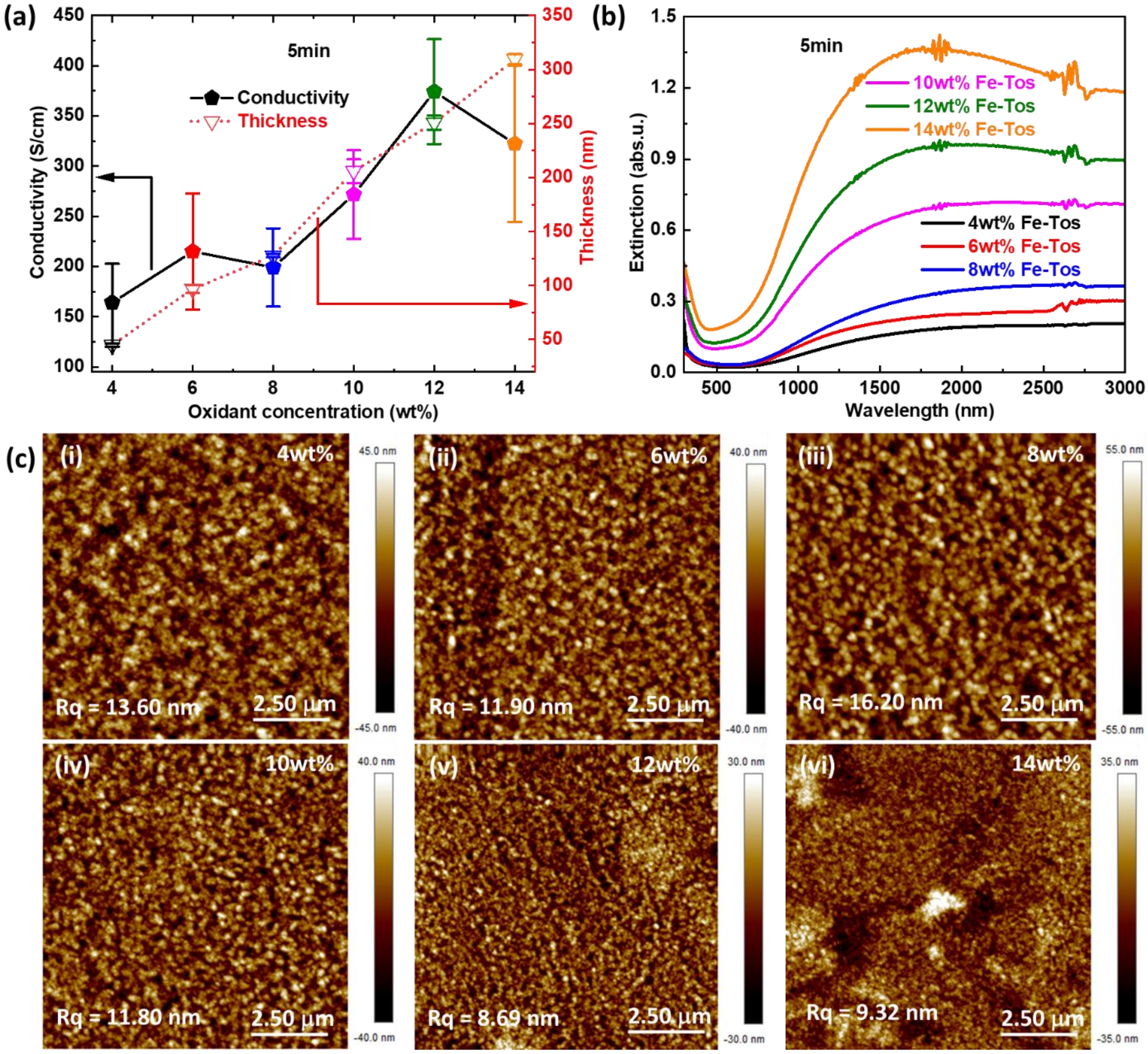

Figure S3: **VPP time (5 min).** (a) Electrical conductivity and thickness variation of pT34bT:Tos with the different oxidant concentration. Here, the temperature for VPP remains constant viz. 60 °C. (b) Extinction spectra of same films recorded in UV-Vis-IR region. (c) (i-vi) AFM images of VPP pT34bT:Tos thin films with different oxidant concentration at 60 °C VPP temperature and 5 min VPP time. Based on roughness values (Rq), optimized concentration-based film (12 wt% Fe-Tos) is showing the smooth morphology.

Figure S4: Optimization of VPP process based on 10 min VPP reaction time (pT34bT:Tos film) with absorption profile and topological features

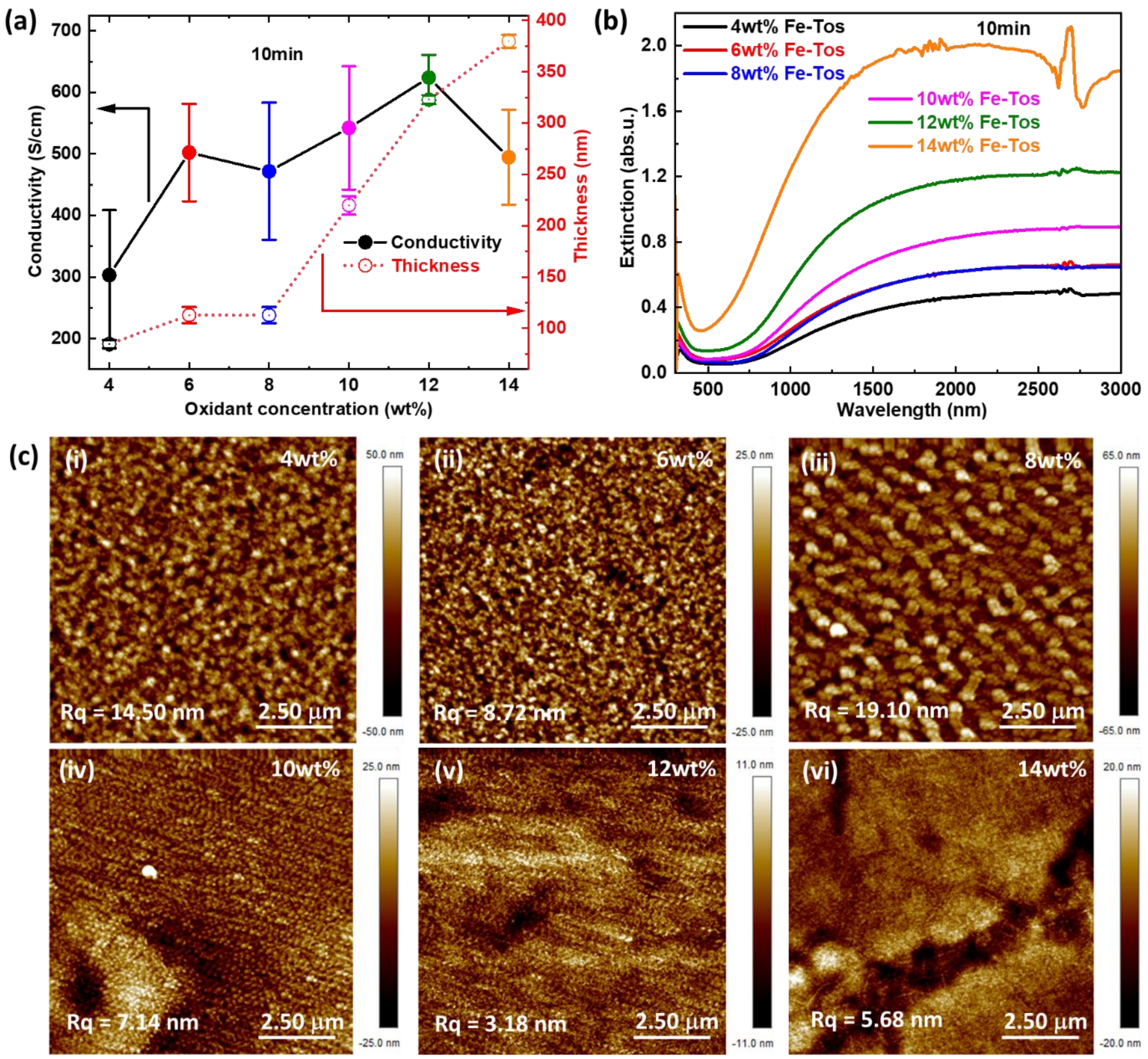

Figure S4: **VPP time (10 min).** (a) Electrical conductivity and thickness tuning of pT34bT:Tos with respect to different oxidant concentration. VPP temperature for VPP remains constant viz. 60 °C. (b) UV-Vis-IR extinction spectra of respective VPP thin films. (c) AFM images (i-vi) disclose the morphological features of VPP pT34bT:Tos thin films with different oxidant concentration at 60 °C VPP temperature and 10 min VPP time. Optimized film (12 wt% Fe-Tos) reveals the smooth structures with lower roughness value (3.18 nm).

Figure S5: Optimization of VPP process based on 15 min VPP reaction time (pT34bT:Tos film) with absorption profile and morphological features

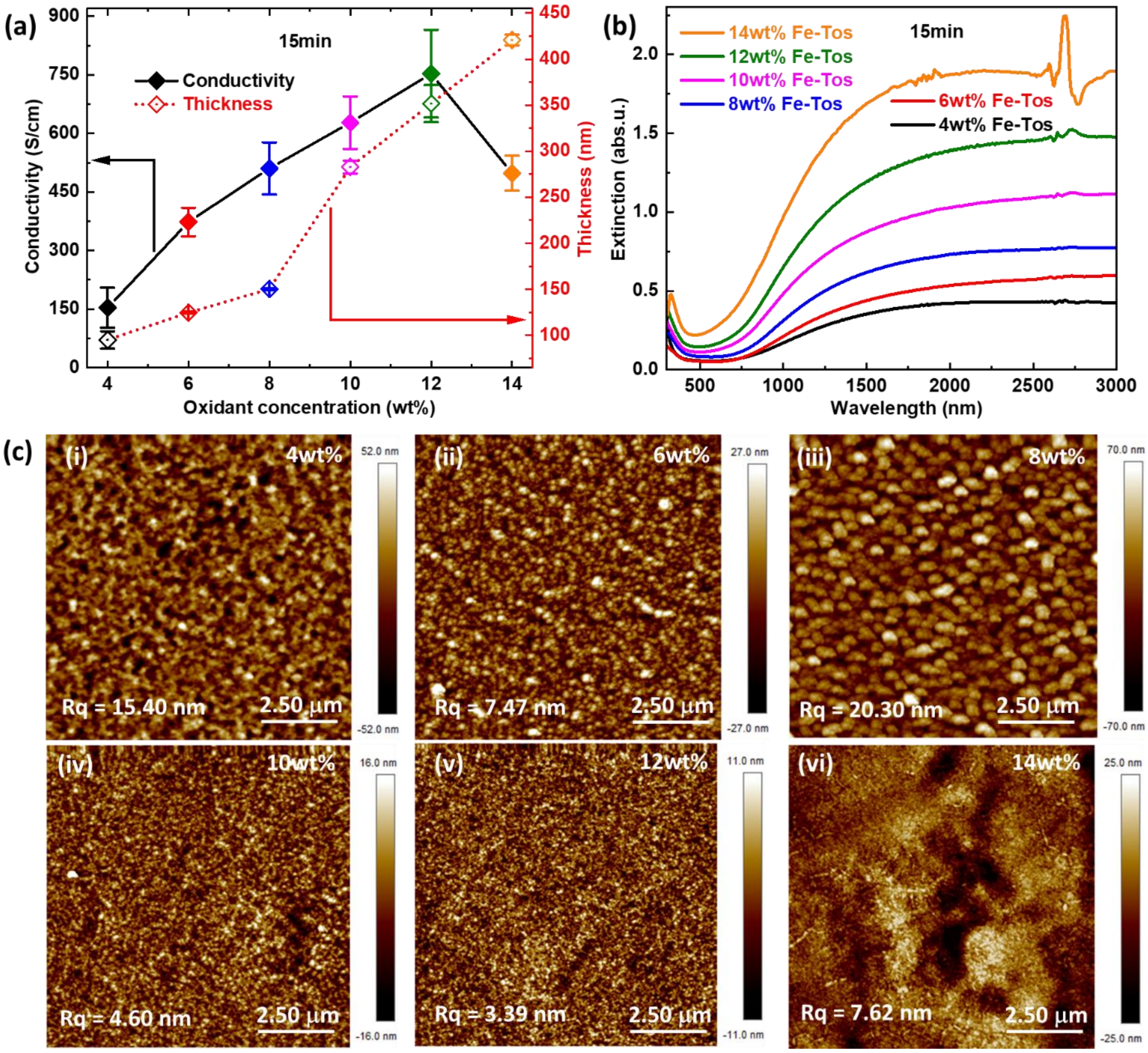

Figure S5: **VPP time (15 min).** (a) Electrical conductivity and film thickness variation of pT34bT:Tos with respect to different oxidant concentration. VPP temperature for VPP process remains constant viz. 60 °C. (b) UV-Vis-IR extinction spectra of respective VPP thin films. (c) AFM (i-vi) images show the morphological features of VPP pT34bT:Tos thin films with different oxidant concentration at 60 °C VPP temperature and 10min VPP time. Optimized VPP process based on Fe-Tos concentration (12 wt%), VPP reaction temperature (60 °C) and VPP time (15 min) show the growth of well oriented and aligned polymer film with roughness value of 3.39 nm.

Figure S6: Optimization of VPP process based on 30 min VPP reaction time (pT34bT:Tos film) with absorption profile and AFM analysis

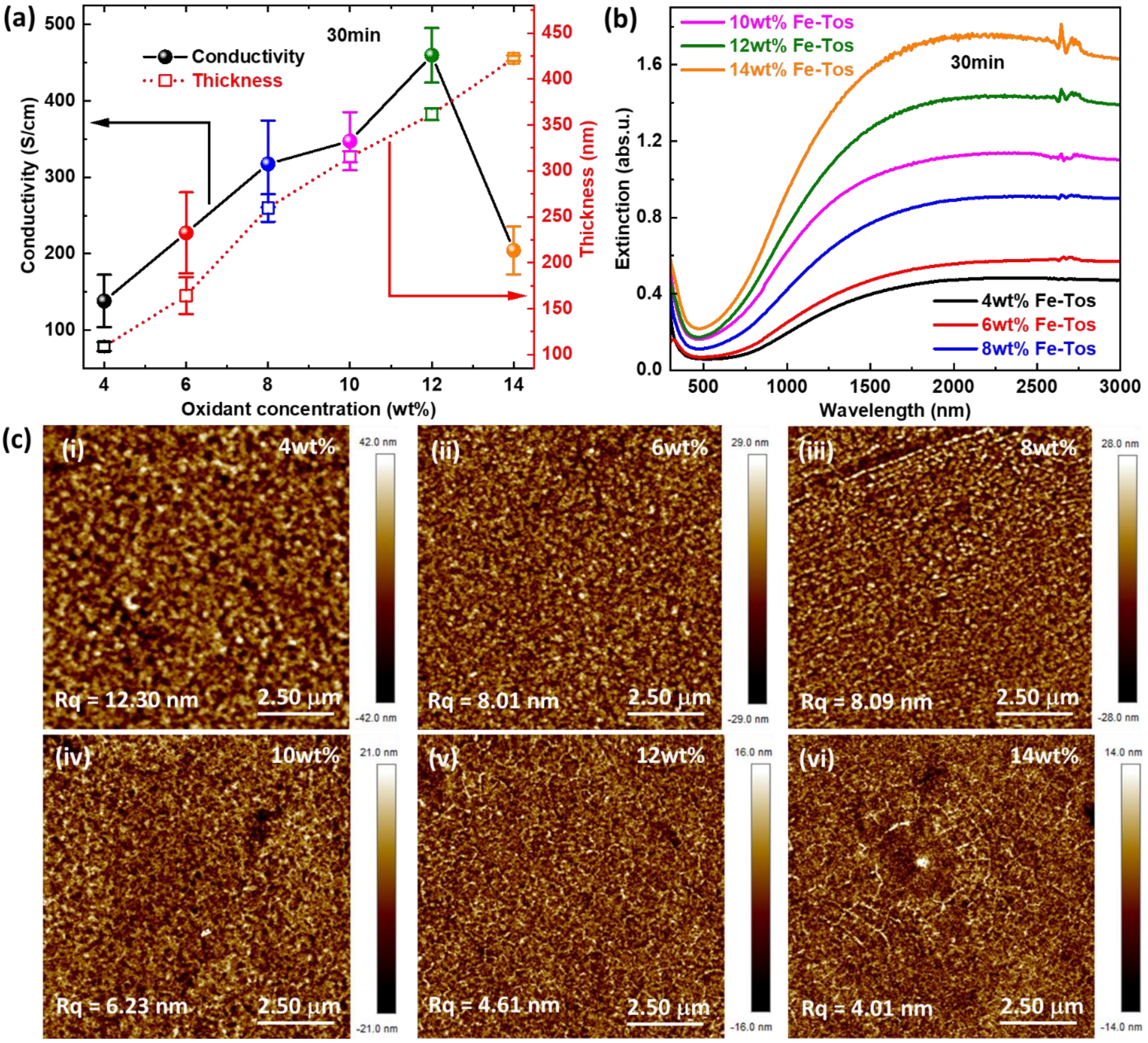

Figure S6: **VPP time (30 min).** (a) Electrical conductivity and thickness modulation of pT34bT:Tos with respect to different oxidant concentration. VPP temperature for VPP remains constant viz. 60 °C. (b) UV-Vis-IR extinction spectra of respective VPP thin films. (c) (i-vi) AFM images divulge the structural features of as grown VPP pT34bT:Tos thin films with different oxidant concentration at 60 °C VPP temperature and 30 min VPP time.

Figure S7: Optimization of VPP process based on 50 °C VPP reaction temperature (pT34bT:Tos film) with absorption profile and structural features

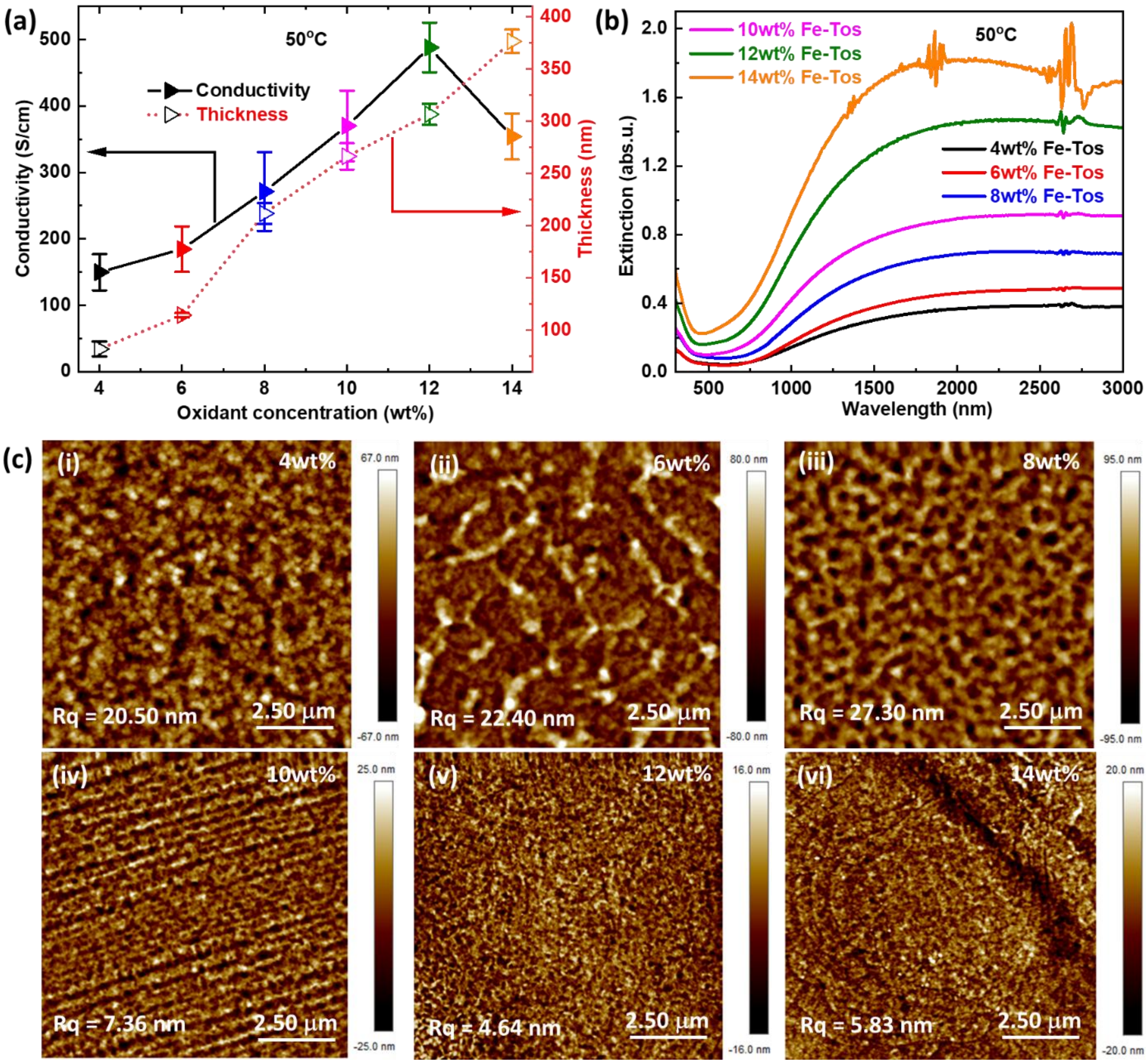

Figure S7: **VPP temperature (50 °C).** (a) Modulation of the electrical conductivity and thickness based on different oxidant concentration pT34bT:Tos thin films. VPP time for VPP remains constant viz. 15 min. (b) UV-Vis-IR extinction spectra of respective VPP thin films. (c) (i-vi) AFM images display the structural features of VPP pT34bT:Tos thin films with different oxidant concentration at 50 °C VPP temperature and 15 min VPP time.

Figure S8: Optimization of VPP process based on 70 °C VPP reaction temperature (pT34bT:Tos film) with absorption profile and morphological features

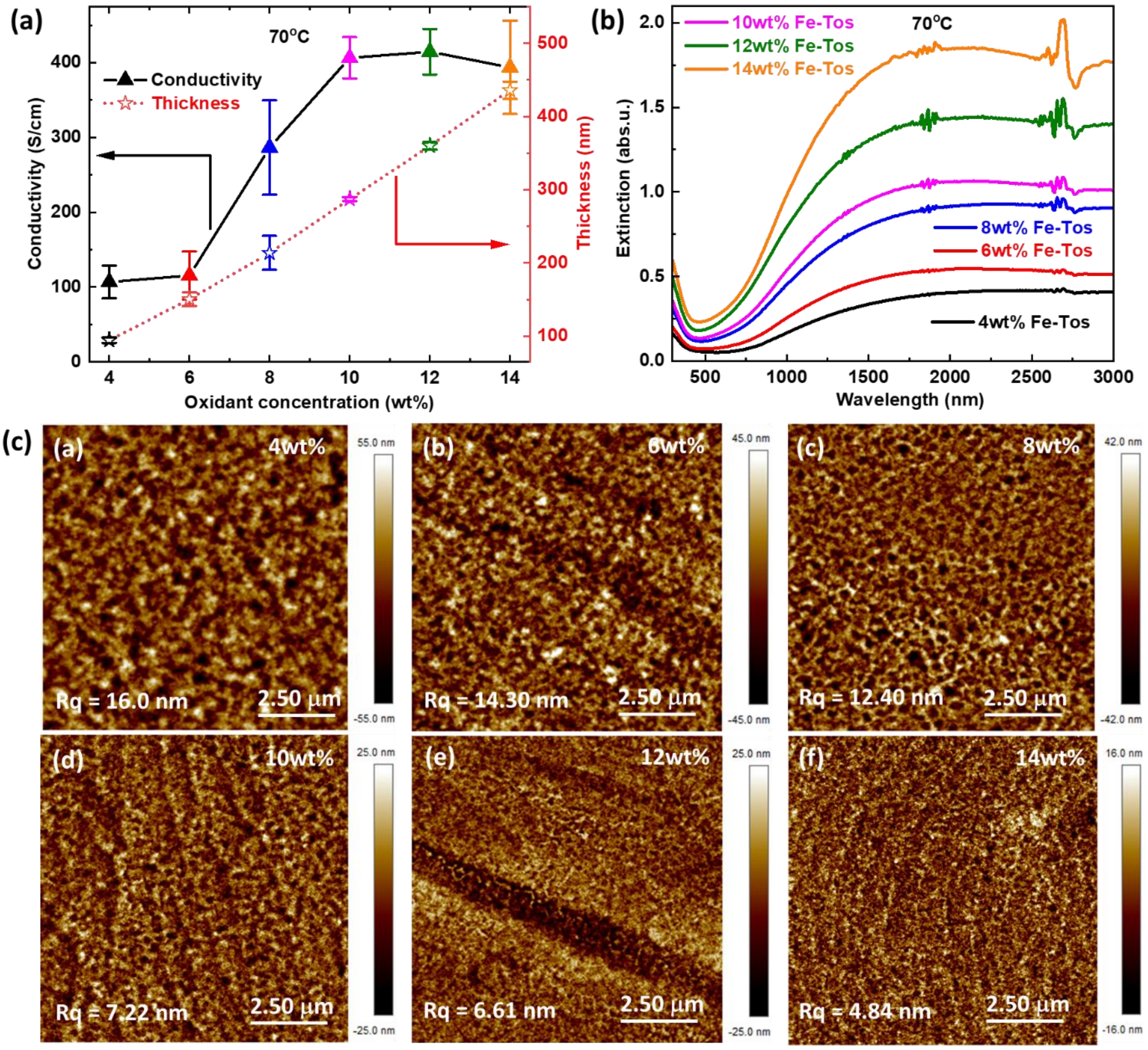

Figure S8: **VPP temperature (70 °C).** (a) Electrical conductivity and thickness tuning of pT34bT:Tos with respect to different oxidant concentration. VPP time for VPP remains constant viz. 15 min. (b) UV-Vis-IR extinction spectra of respective VPP thin films. (c) (i-vi) AFM images divulge the structural features of as grown pT34bT:Tos thin films with different oxidant concentration at 70 °C VPP temperature and 15 min VPP time.

Figure S9: Simulated reflectance of pT34bT:Tos-based optical cavities

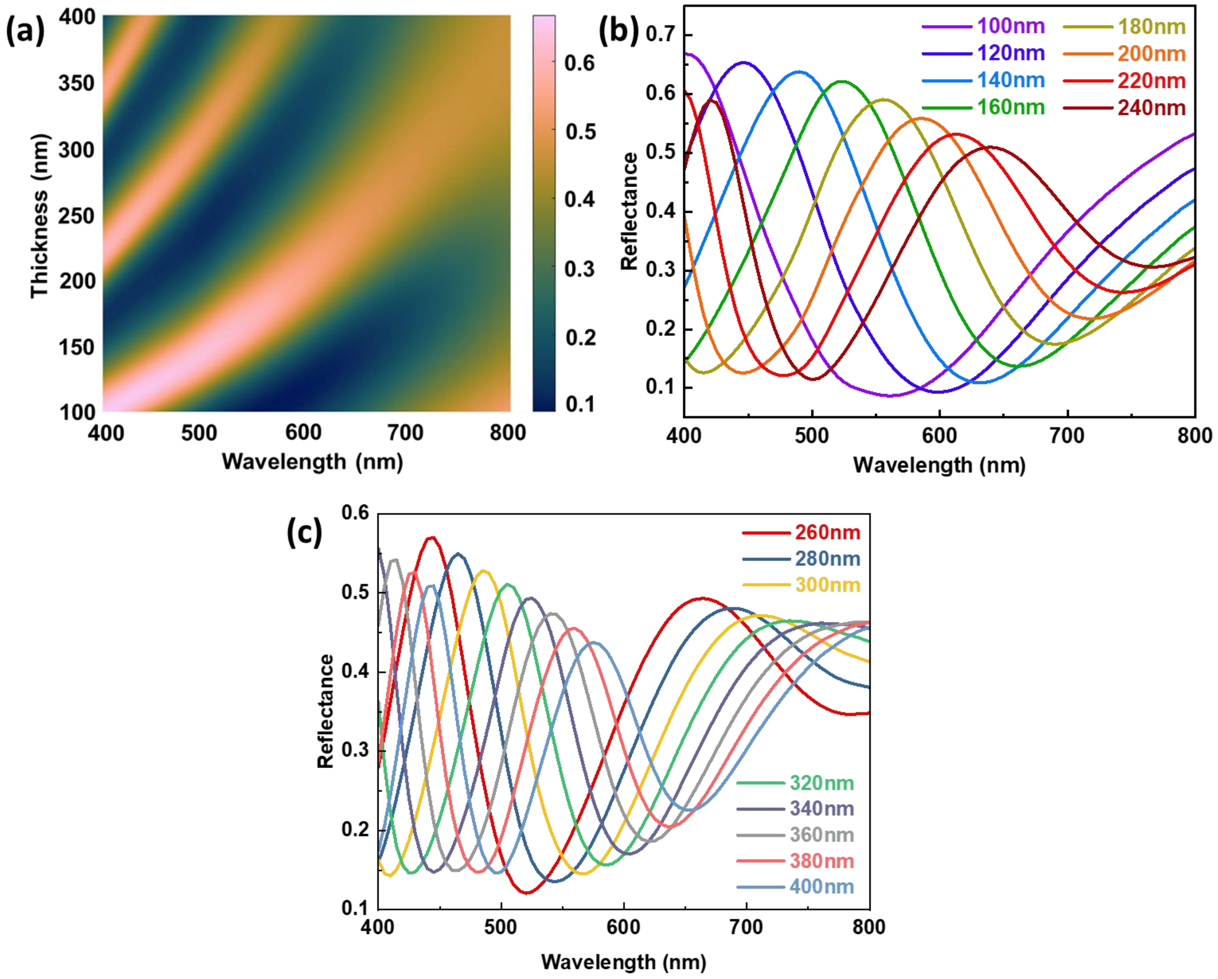

Figure S9: Simulated reflectance of pT34bT:Tos-based optical cavities. (a) Reflectance color map for simulated optical cavities based on Mirror (with 70nm Al, 3nm Cr and 7nm Au) and semi-transparent mirror (with 5nm Cr and 7nm Au) for different thicknesses of the VPP pT34bT:Tos. (b and c) Simulated reflectance spectra of pT34bT:Tos based optical cavities with different polymer thicknesses.

Figure S10: Conductivity and thickness variation of UV patterned pT34bT: Tos thin films

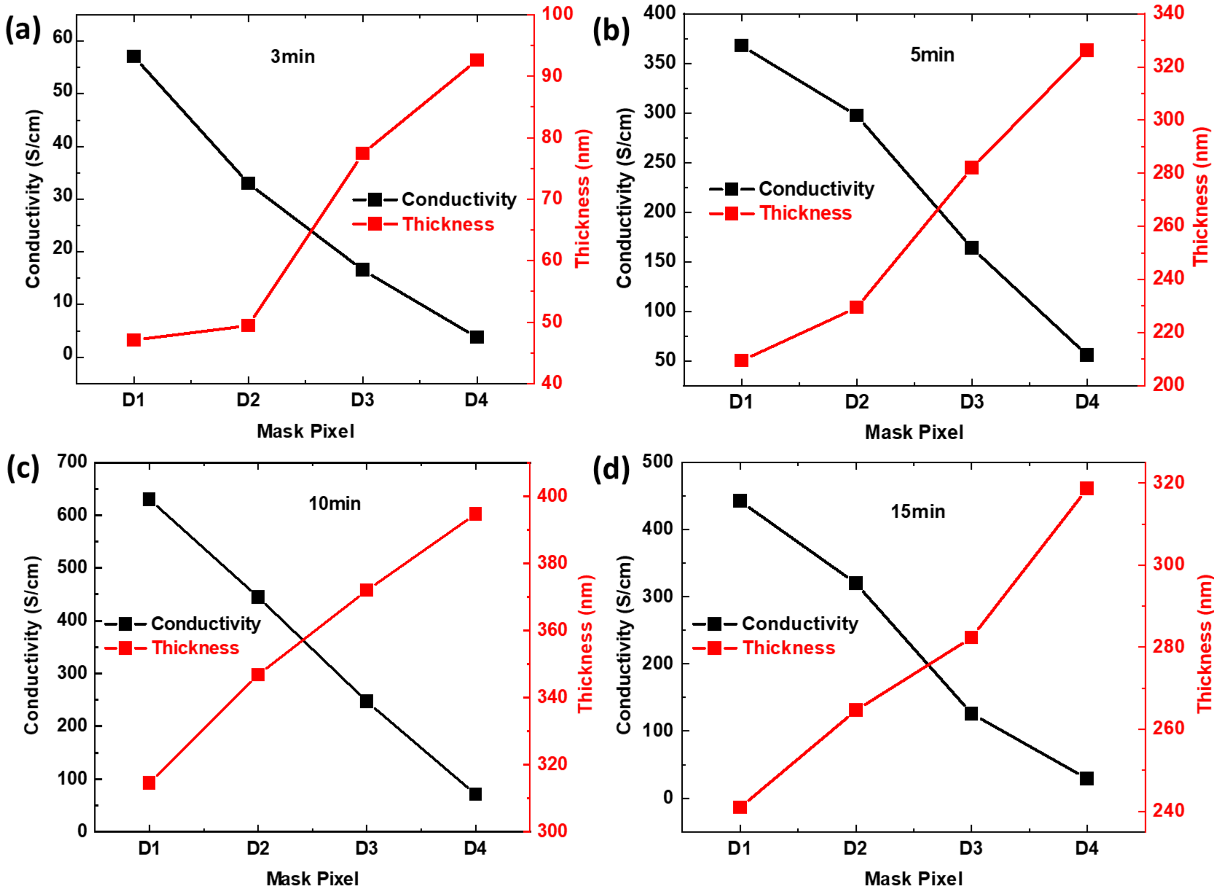

Figure S10: Variation of conductivity and thickness of UV-irradiated pT34bT:Tos. (a-d) Conductivity and thickness of UV patterned pT34bT using UV exposure through the photomask shown in Figure 5b (with increasing UV transmittance for increasing pixel number, D1-D4). The UV exposure time was kept at 20 min, and the polymerization time was varied as: (a) 3 min, (b) 5 min, (c) 10 min, and (d) 15 min. Conductivity of VPP films decreased with increasing UV dose. For all panels, the data related to pixel D3 (which had less uniform thickness) was determined as the average of measurements on several different spots.

Figure S11: Non-uniform effects based on pixel D3

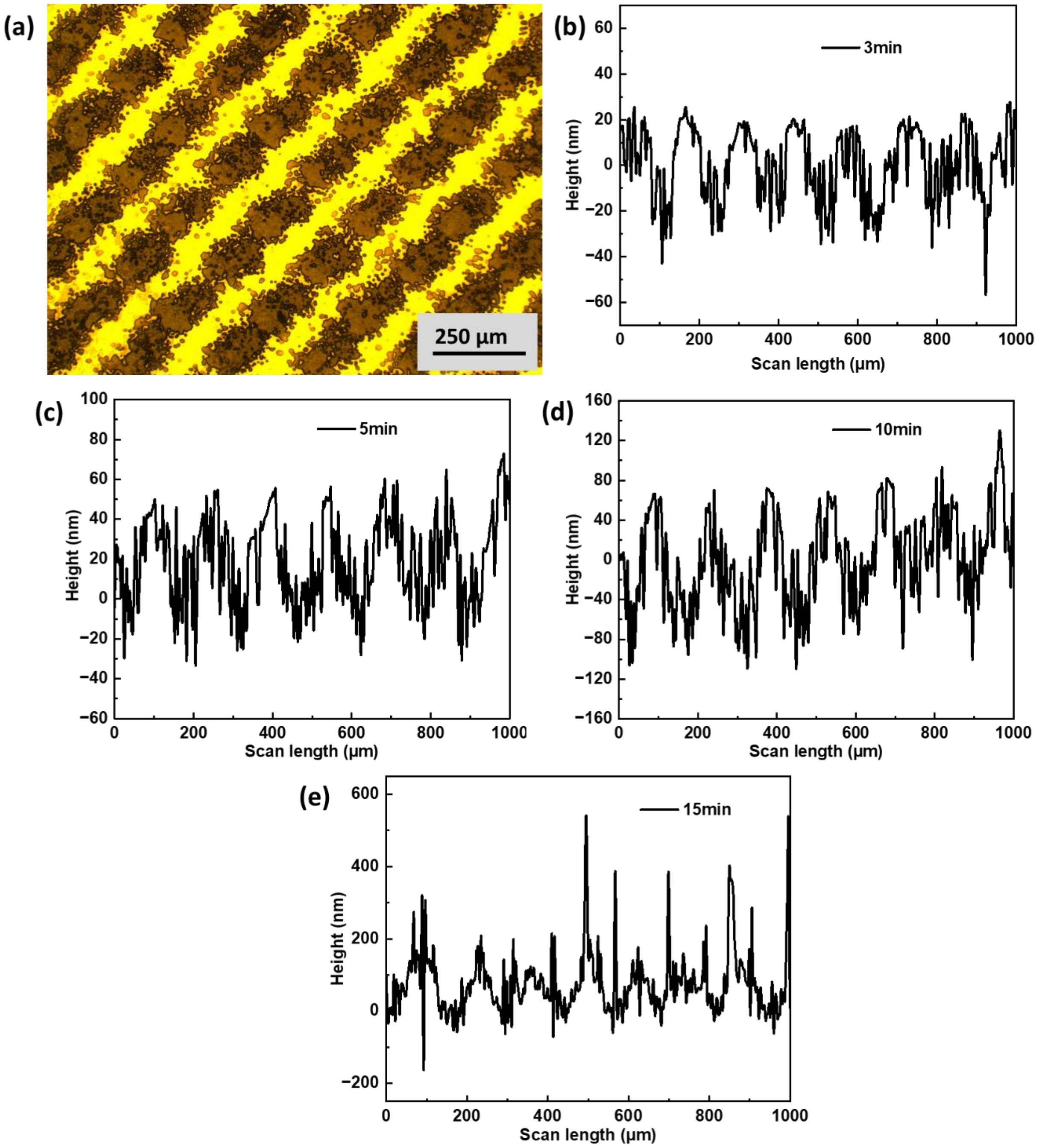

Figure S11: Pixel D3 of photomask. (a) A microscopic image of the D3 pixel of the photomask at 10X magnification shows the presence of a microscale line pattern. (b-e) Profilometry results for pT34bT films deposited with different polymerization times after UV exposure through pixel D3 (20 min), revealing a clear line structure with thickness variations.
